# Large-Scale Evaluation of Maize Germplasm for Low-Phosphorus Tolerance

**DOI:** 10.1371/journal.pone.0124212

**Published:** 2015-05-04

**Authors:** Hongwei Zhang, Ruineng Xu, Chuanxiao Xie, Changling Huang, Hong Liao, Yunbi Xu, Jinxiang Wang, Wen-Xue Li

**Affiliations:** 1 Institute of Crop Science, National Key Facility of Crop Gene Resources and Genetic Improvement, Chinese Academy of Agricultural Sciences, Beijing, China; 2 State Key Laboratory for Conservation and Utilization of Subtropical Agro-Bioresources, South China Agricultural University, Guangzhou, China; 3 Root Biology Center, College of Agronomy, South China Agricultural University, Guangzhou, China; 4 International Maize and Wheat Improvement Center (CIMMYT), El Batán, Texcoco, Mexico; National Institute of Plant Genome Research (NIPGR), INDIA

## Abstract

Low-phosphorus (LP) stress is a global problem for maize production and has been exacerbated by breeding activities that have reduced the genetic diversity of maize. Although LP tolerance in maize has been previously evaluated, the evaluations were generally performed with only a small number of accessions or with samples collected from a limited area. In this research, 826 maize accessions (including 580 tropical/subtropical accessions and 246 temperate accessions) were evaluated for LP tolerance under field conditions in 2011 and 2012. Plant height (PH) and leaf number were measured at three growth stages. The normalized difference vegetation index (NDVI) and fresh ear weight (FEW) were also measured. Genetic correlation analysis revealed that FEW and NDVI were strongly correlated with PH, especially at later stages. LP-tolerant and -sensitive accessions were selected based on the relative trait values of all traits using principal component analysis, and all the 14 traits of the tolerant maize accessions showed less reduction than the sensitive accessions under LP conditions. LP tolerance was strongly correlated with agronomic performance under LP stress conditions, and both criteria could be used for genetic analysis and breeding of LP tolerance. Temperate accessions showed slightly better LP tolerance than tropical/subtropical ones, although more tolerant accessions were identified from tropical/subtropical accessions, which could be contributed by their larger sample size. This large-scale evaluation provides useful information, LP-tolerant germplasm resources and evaluation protocol for genetic analysis and developing maize varieties for LP tolerance.

## Introduction

As a worldwide food and feed crop, maize ranks first in total production among major staple cereals [1]. Maize yield, however, is frequently threatened by various abiotic stresses, including low-phosphorus (LP) stress [2]. At the same time, LP stress is made worse due to P fixation by inorganic and organic materials in most soils, low resources input in underdeveloped nations, and soil degradation [2, 3]. It is therefore imperative to develop LP-tolerant maize varieties.

To adapt to LP environments, plants have evolved elaborate systems for P scavenging, acquisition, and recycling [2, 4], which are generally called phosphate starvation responses (PSRs). PSRs in plants include the remodeling of root system architecture, the secretion of organic acids and acid phosphatases, the remobilization of internal P, and the decrease of biomass and yield [5, 6]. Researchers have determined that LP tolerance is related to multiple PSRs that are manifested in plant traits [5, 7]. For example, plant height (PH) and shoot dry weight of rice were inhibited under LP conditions, and these traits were therefore used to measure tolerance to LP stress in rice [8]. Maize inbred lines tolerant to LP stress were found to have greater activity of secreted acid phosphatases and higher root organic acid content than sensitive inbred lines under LP conditions [7, 9]. Thus PSRs of crops provide the foundation for measuring LP tolerance and for selecting LP-tolerant germplasm resources.

Exotic germplasm of crop plants often contains favorable alleles. Wild wheat, for example, contains a NAC transcription factor that accelerates senescence and increases nutrient remobilization to facilitate the flow of nutrients from old tissues to developing seeds, while the modern wheat allele is non-functional [10]. In maize, the favorable allele near a short upstream region of the *Tb1* gene, which can repress axillary organ development, has been fixed in tropical and northern flint accessions [11]. In addition, genotyping has revealed that tropical maize has greater genetic diversity and contains more rare alleles than temperate maize [12, 13]. Rare alleles of functional genes were reported to increase maize yield and carotene content [14, 15]. Therefore, introducing favorable alleles from tropical into temperate maize has been a major breeding goal in the USA [16] and China [17].

Although LP tolerance in maize has been previously evaluated, the evaluations were generally performed with only a small number of accessions or with samples collected from a limited area, or evaluated under greenhouse conditions [18–20] Therefore, it is necessary to evaluate a more diverse set of maize germplasm resources in order to identify and select maize materials with increased tolerance to LP stress. In this study, 826 maize accessions (representing tropical/subtropical and temperate germplasm) from CIMMYT and China were evaluated under both LP and normal-phosphorus (NP) field conditions. Our objectives were to: (1), identify maize plant traits that could be used to select for LP tolerance; (2), identify accessions that could be used for enhancing LP tolerance; (3), compare LP tolerance of the tropical/subtropical ecotype and the temperate ecotype. Among the selected 41 tolerant accessions and 41 sensitive accessions, more belonged to the tropical/subtropical ecotype than the temperate ecotype. The identified LP-tolerant and-sensitive accessions will be useful for improving LP tolerance of maize varieties and for conducting genetic and biological analysis of LP tolerance in maize.

## Materials and Methods

### Plant materials

A total of 826 accessions (S1 Table) were used in the field experiment, which was first conducted in 2011 and then repeated with some modification in 2012. The accessions include recombinant inbred lines (RILs), introgression lines, and inbred lines. The two RIL populations were C5 RIL (Ac7643×Ac7729/TZSRW, containing 170 lines) and C6 RIL (CML444×Malawi, containing 193 lines), both of which were developed from tropical inbred lines at International Maize and Wheat Improvement Center (CIMMYT). Ac7643 was more tolerant to LP stress than Ac7729/TZSRW (S1 Table). The IL population was developed using eight Chinese elite inbred lines (Chang7-2, Qi319, Dan340, Dan598, Dan599, He344, Dong91, and Ye478) as recurrent parents and 30 inbred lines from CIMMYT and different ecological zones in China as donors, including CML098, CML193, Tie84, Han21, and Zheng58. The IL population was derived from three different backcross generations (BC_2_F_4_, BC_2_F_5_ and BC_4_F_3_), with159 lines in total. The inbred lines included 217 tropical/subtropical lines from CIMMYT and 87 temperate inbred lines from the Chinese Academy of Agricultural Sciences (CAAS). For simplicity, the tropical/subtropical and temperate inbred lines are designated as the “Trop” and “Temp” populations, respectively.

### Nursery and field management

The maize accessions were evaluated at South China Agricultural University experimental station (113°83′E, 23°31′N), Zengcheng, Guangdong in 2011 and 2012. The station has an annual precipitation of 1732 mm [21]. The 2011 experiment included LP and NP treatments in nursery field, while the 2012 experiment included an LP treatment in nursery field and an NP treatment in the field.

The basic chemical characteristics of the soil, sampled at 0 to 20 cm depth before fertilizer application in 2011 and 2012, were listed in Table 1. More P fertilizers were applied to the NP field or nursery in order to enlarge the differences between the two treatments. Regarding fertilization in 2011, N, P_2_O_5_, and K_2_O were applied to the NP plots before seeds were sown at 70, 69, and 90 kg/ha, respectively. The same amounts of fertilizer except the P_2_O_5_ were applied to the LP plots in 2011. In addition, 25 μg KH_2_PO_4_ was applied near the root of every seedling in the LP plots at 36 and 46 days after planting (DAP) in 2011 because of the severe growth inhibition by LP stress. Regarding fertilization in 2012, N, P_2_O_5_, and K_2_O were applied to the NP plots at 72, 120, and 100 kg/ha, respectively, and were applied to the LP plots at 72, 72, and 100 kg/ha, respectively. The plots were irrigated every two weeks, and the soil was kept moist throughout the growing season.

**Table 1:** Basic chemical characteristics of the soil (0 to 20 cm depth) used to evaluate 826 maize accessions in Guangzhou, China.

Year	Treatment	pH	Organic matter	Available P (mg/kg)	Alkaline N (mg/kg)	Available K (mg/kg)
2011	LP	5.01	0.99%	4.66	14.35	55.35
	NP	5.08	1.08%	5.82	16.94	71.20
2012	LP	4.89	1.16%	4.23	21.84	63.20
	NP	5.51	6.63%	154.10	97.44	86.60

### Trait evaluation

The maize accessions were evaluated under the two P conditions in both years in an alpha (0, 1) lattice design [22], with two replications for each treatment. Seeds were sown on 24 September 2011 and on 10 September 2012. Each line was sown in a two-row plot, with five plants per row, and with 25 cm between plants and between rows. One row of each plot was thinned so that row spacing was increased to 50 cm at 35 DAP. PH and leaf number (LN) were recorded for four plants in each plot at 23 DAP, and for three plants at 53 and 63 DAP. In 2011, the normalized differential vegetation index (NDVI), which was considered as a stable indicator of shoot biomass [23], was measured with a GreenSeeker crop sensor (Trimble Navigation Limited, USA) at 51 DAP. Fresh ear weight (FEW) for three plants in each plot was determined in 2012, and the average FEW was calculated consequently. The inbred line-Zheng58, which didn’t show obvious response to LP stress [20] and was demonstrated in our preliminary experiments (data not shown), was used as a reference line to adjust for environmental variation. Zheng58 was planted in every 20 plots in 2012, and all traits were adjusted relative to the means of Zheng58 traits.

### Data analysis

Means, standard deviations (SDs), and coefficients of variance (CVs) were calculated with Excel 2007. ANOVAs, genetic correlation, and broad-sense heritability analysis were performed using SAS [24] following the lattice design [25].

The relative trait value is the most commonly used index for measuring LP tolerance [26]. To determine a relative trait value, the trait value for the LP treatment was divided by the corresponding trait value for the NP treatment. The relative trait values of all traits were standardized with the means set at 0 and the SDs set at 1. The standardized trait values were then subjected to principal component (PC) analysis using SAS [24]. PCs whose eigen-values were ≥1 were retained [26] and used to calculate an LP tolerance index (LPTI) following the formula:

$$\text{LPTI = }\sum_{\text{i=1}}^{\text{n}}\text{PCi}*\text{CRi}$$ (1)

where n is the number of PCs with eigen-values ≥1, and CR (contribution rate) is the rate for variation of all relative trait values. LPTI were used to select the 41 most tolerant and 41 most susceptible accessions, each accounting for ~5% of the tested accessions.

The traits were assigned to two groups: biomass traits and leaf traits. Biomass traits reflect the amount of dry materials accumulated by plants or reproductive organs, and include PH, NDVI, and FEW. The sole leaf trait is LN, which reflects the rate of leaf development. To compare the selection results based on the two trait groups, each trait group was used to calculate LPTIs. The LPTIs calculated using biomass and leaf traits were designated LPTI_bm and LPTI_lf, respectively. Similarly, the results of selection performed at different stages were also compared. The traits measured at 23 DAP, before the internodes had begun to elongate, were used as early stage traits. The traits measured at >51 DAP, when the tassels of most accessions had emerged, were used as late-stage traits. The LPTIs calculated using early and late-stage traits were designated LPTI_el and LPTI_lt, respectively.

The accessions showing better trait performance under LP conditions should be desirable for developing LP-tolerant hybrids [26]. To quantify the trait performance under LP conditions, the traits under LP conditions were used to calculate an LP performance index (LPPI). To calculate the LPPI, the standardized trait value for each trait under LP conditions was subjected to PC analysis, and the PCs with eigen-values ≥1 were then used to calculate LPPI with the formula (1). The accessions could be classified into four extreme groups based on LPPI and LPTI, these groups were the tolerant and good-performance group (TG), the sensitive and good-performance group (SG), the tolerant and poor-performance group (TP), and the sensitive and poor-performance group (SP).

## Results

### Phenotypic variation of traits under NP and LP conditions

**Fig 1:**
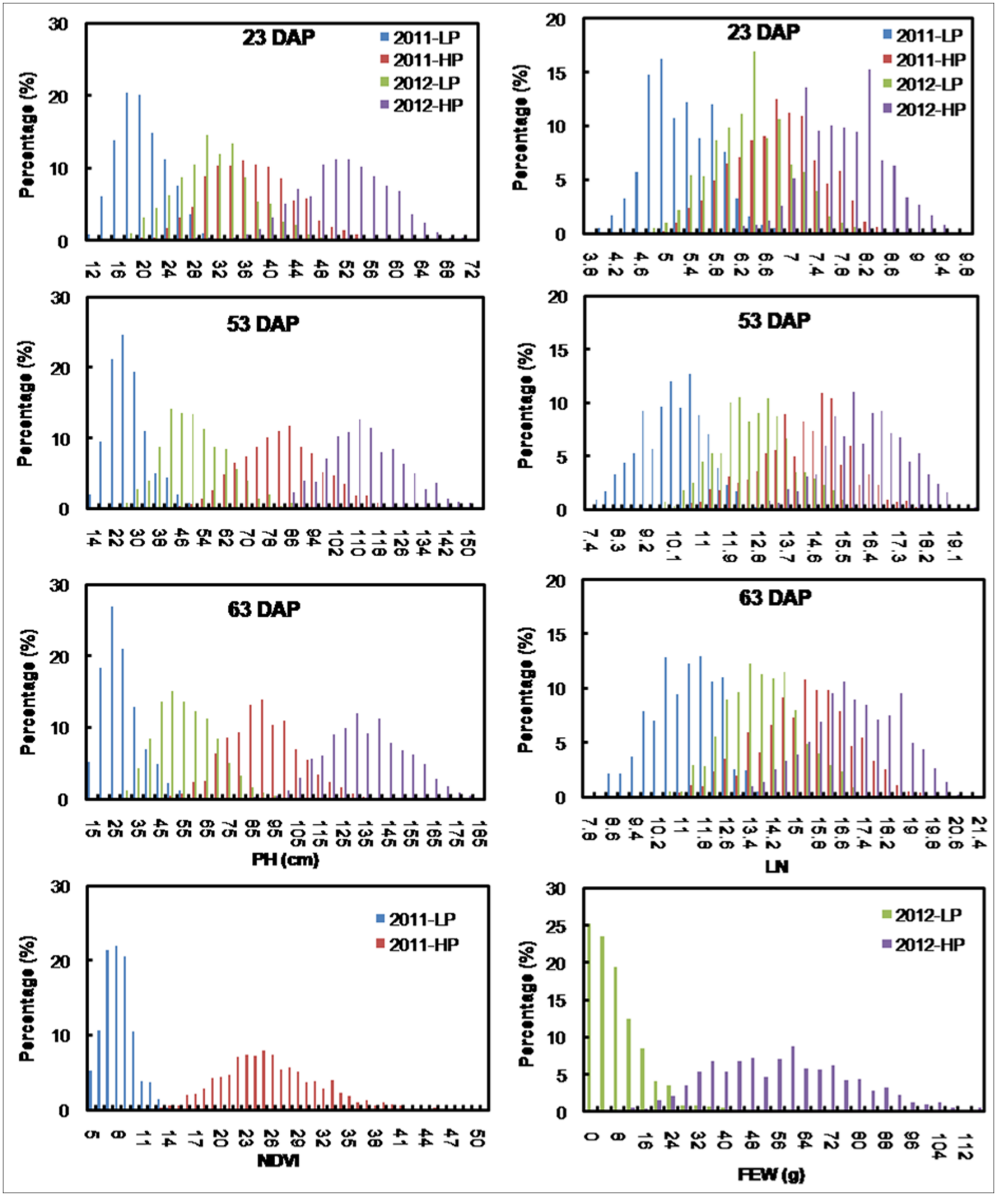
Distribution of all traits measured for 826 maize accessions. PH: plant height; LN: leaf number; NDVI: normalized difference vegetation index; FEW: fresh ear weight; DAP: days after planting; LP: low-P; NP: normal-P; Percentage: the number of accessions in each interval divided by the total number of accessions.

**Fig 2:**
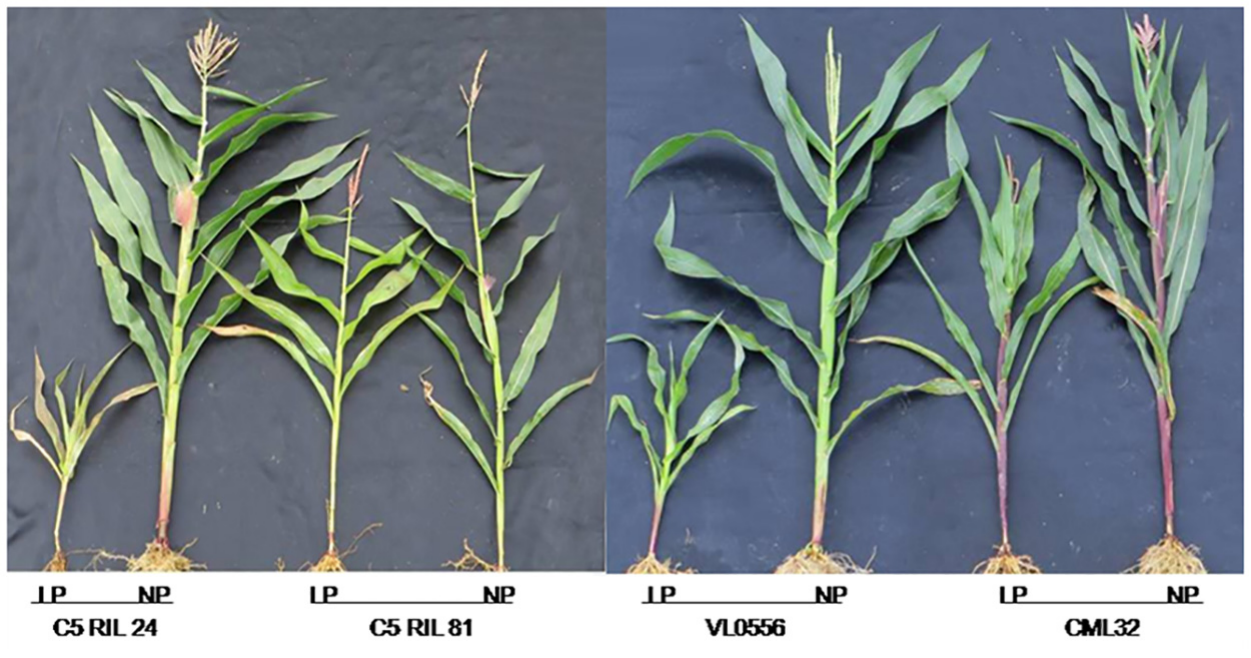
Responses of LP tolerant and susceptible accessions to LP stress. Photos were taken at 57 DAP in 2012.

The phenotypic means in NP plots were generally larger in 2012 than in 2011, perhaps because of the significant differences in soil nutrient content before fertilization (Table 1) and the amounts of fertilizers applied. The phenotypic means in LP plots were also larger in 2012 than in 2011, perhaps because of the higher rate of P fertilization in 2012. Trait performance was better with the NP than with the LP treatment in both years, indicating that the phenotypic differences between the two treatments were mainly caused by different P levels in the soil or different amounts of P fertilizer applied (Figs 1 and 2; Table 1).

ANOVAs revealed that the variation among the genotypes was significant for all the traits observed across developmental stages. At the same time, the differences between the two treatments, and the interaction between treatment and genotype were also significant for all traits (Table 2). The SDs were lower under LP conditions than under NP conditions for all traits, suggesting that the phenotypic variation for all the traits increased under NP conditions (Table 2). The CVs were generally lower in 2012 than in 2011, which might be explained by the phenotypic adjustment that was done for 2012 data using the reference inbred line. At the same time, the SDs and CVs were lower for LN than for PH, NDVI, and FEW, indicating that LN was the most stable trait. The LP treatment had the greatest impact on FEW, followed by NDVI and PH, and the LP treatment had the smallest impact on LN, as indicated by its high relative trait value (Table 2).

**Table 2:** Means and heritabilities for the tested traits under different P conditions.

Traits	Year	Sample size		Mean ± SD		Relative trait value	CV(%)		Heritability		ANOVA			
		LP	NP	LP	NP		LP	NP	LP	NP	Geno	Treat	Eco	Geno*Treat
PH23	2011	826	826	21.2±3.8	38.1±7.0	0.56	17.78	18.28	0.89	0.64	***	***	ns	***
	2012	826	826	32.4±6.1	52.4±7.1	0.62	18.89	13.56	0.58	0.70	***	***	ns	***
PH53	2011	826	826	29.7±7.1	85.0±14.5	0.35	23.89	17.02	0.70	0.54	***	***	ns	***
	2012	826	826	53.0±11.6	114.8±14.0	0.46	21.94	12.18	0.41	0.74	***	***	ns	***
PH63	2011	826	826	31.5±8.6	92.9±15.5	0.34	27.22	16.67	0.71	0.56	***	***	ns	***
	2012	826	826	58.9±13.0	137.0±17.5	0.43	22.06	12.76	0.46	0.81	***	***	ns	***
LN23	2011	826	826	5.4±0.5	6.9±0.7	0.79	9.84	10.03	0.71	0.67	***	***	ns	***
	2012	826	826	6.5±0.6	7.9±0.6	0.81	9.63	7.98	0.54	0.78	***	***	ns	***
LN53	2011	814	826	10.3±1.0	14.5±1.4	0.71	9.81	9.91	0.72	0.69	***	***	ns	***
	2012	826	826	12.8±1.2	16.4±1.3	0.79	9.32	7.69	0.58	0.83	***	***	ns	***
LN63	2011	808	826	11.4±1.1	15.6±1.7	0.73	9.96	10.62	0.71	0.66	***	***	ns	***
	2012	826	826	14.1±1.3	17.3±1.6	0.82	9.24	9.03	0.62	0.87	***	***	*	***
NDVI(%)	2011	821	824	8.7±1.7	26.5±5.9	0.33	19.98	22.27	0.53	0.39	***	***	**	***
FEW	2012	826	825	10.2±8.1	59.4±20.7	0.17	81.18	34.93	0.40	0.74	***	***	ns	***

Abbreviations: PH: plant height; LN: leaf number; NDVI: normalized difference vegetation index; FEW: fresh ear weight; SD: standard deviation; CV: coefficient of variance; ANOVA: analysis of variance; LP: low-P; NP: normal-P; Geno: genotypes; Treat: treatments; Eco: ecotype; Geno×Treat: interaction between genotypes and treatments. The numbers after the trait abbreviations indicate the number of days after planting.

Note: relative trait value was calculated by dividing the trait value under NP condition by the corresponding trait value under LP condition; lower relative trait values indicate greater sensitivity to LP stress. ***, **, and * indicate significance at 0.001, 0.01, and 0.05 levels, respectively; ns: not significant.

Heritability was highest for LN (mean 0.70, range 0.54–0.87), followed by PH (mean 0.64, range 0.41–0.89). The heritability of FEW under LP conditions was lower than under NP conditions, perhaps as a result of the severe stress. Heritability was lowest for NDVI (0.39 for LP and 0.53 for NP), and these values were lower than those reported previously [23]. The small number of plants per row in our experiment might have contributed to the low heritability values for NDVI. Heritability did not significantly differ (P>0.05) between the two treatments. In most cases, traits were highly heritable under both conditions, indicating that the traits measured are useful for the evaluation of LP tolerance.

### Phenotypic performance of traits and their genetic correlation

Both PH and LN increased with growth stage, and increased at higher rates with plant development under NP conditions than under LP conditions (Fig 1; Table 2). The largest differences were observed at 63 DAP for PH and at 53 DAP for LN in both years, indicating that phenotypic differences associated with genetic variation were increasingly expressed and became more distinct in the later stages (Table 2).

Genetic correlation analysis revealed that: (1), NDVI and FEW were strongly correlated with PH and LN (especially at the last two stages) in most cases, suggesting that PH and LN (especially PH) at later stages contributed more to biomass and fresh ear weight; (2), the trait correlation between the two P treatments was strong for most traits, which might be caused by some genetic factors that took effect under both P conditions; (3), PH at different stages was strongly correlated with each other, implying that PH at different stages was controlled by some common genes, and this was also true for LN; (4), the correlation between PH and LN at the same stage was usually strong, although these two traits represent two different trait groups (biomass and leaf traits). The frequent occurrence of a significant correlation between PH and LN suggested that leaf emergence might occur with an increase in PH (Table 3).

**Table 3:** Genetic correlations among some tested traits.

		LP								NP						
		PH23	PH53	PH63	LN23	LN53	LN63	FEW	NDVI	PH23	PH53	PH63	LN23	LN53	LN63	NDVI
LP	PH23	1	0.59	0.51	0.28				0.39	0.56						
	PH53	0.64	1	0.93		0.29			0.67		0.31					
	PH63	0.58	0.91	1			0.31		0.67			0.31				
	LN23	0.44			1	0.57	0.57		0.24				0.46			
	LN53		0.53		0.72	1	0.82		0.41					0.52		
	LN63			0.52	0.65	0.88	1		0.40						0.36	
	FEW	0.32	0.50	0.55	0.15	0.31	0.35	1								
	NDVI								1							0.19
NP	PH23	0.47								1	0.70	0.70	0.58			0.54
	PH53		0.26							0.56	1	0.93		0.52		0.74
	PH63			0.26						0.45	0.80	1			0.47	0.71
	LN23				0.50					0.30			1	0.69	0.66	0.43
	LN53					0.45					0.20		0.64	1	0.88	0.52
	LN63						0.49					0.16	0.52	0.83	1	0.50
	FEW							0.32		0.15	0.20	0.39	0.01	0.10	0.20	

Note: The numbers after the trait abbreviations indicate the number of days after planting. Correlation coefficients in 2011 are listed in the upper-right quadrant, and those in 2012 are listed in the lower-left quadrant. Correlation coefficients ≥ 0.07, 0.09, and 0.11 were significant at 0.05, 0.01, and 0.001 levels, respectively.

### Selection of LP-tolerant and-sensitive accessions

To avoid underestimating those traits that were greatly influenced by LP stress, standardized relative trait values for all traits were used to calculate LPTI, which was further used to classify the accessions. Because LPTI was calculated from the standardized relative trait values for all traits, we analyzed the correlation between LPTI and the relative trait value for each trait to confirm LPTI credibility and reliability. LPTI was strongly correlated with the relative trait value for each trait (S2 Table), supporting the feasibility of using LPTI to classify LP tolerance of the germplasm.

Among the 41 sensitive accessions that were selected based on LPTI, 20, 1, 8, 11, and 1 accessions were from C5, C6, IL, Trop, and Temp populations, respectively. Among the 41 tolerant accessions that were selected based on LPTI, 2, 24, 4, 8, and 3 accessions were from C5, C6, IL, Trop, and Temp populations, respectively (S3 Table). Traits were compared between the sensitive and tolerant groups to confirm the reliability of selection. As shown in S1 Fig, trait performance of the sensitive group showed the greatest reduction when the LP stress was imposed. At 23 DAP in 2011, for example, PH was 56% lower under LP vs. NP for the sensitive group and was 28% lower for the tolerant group. Similar trends were observed for the other traits (S1 Fig), which validated our selection results.

### Trait performance under LP conditions and LP tolerance

**Fig 3:**
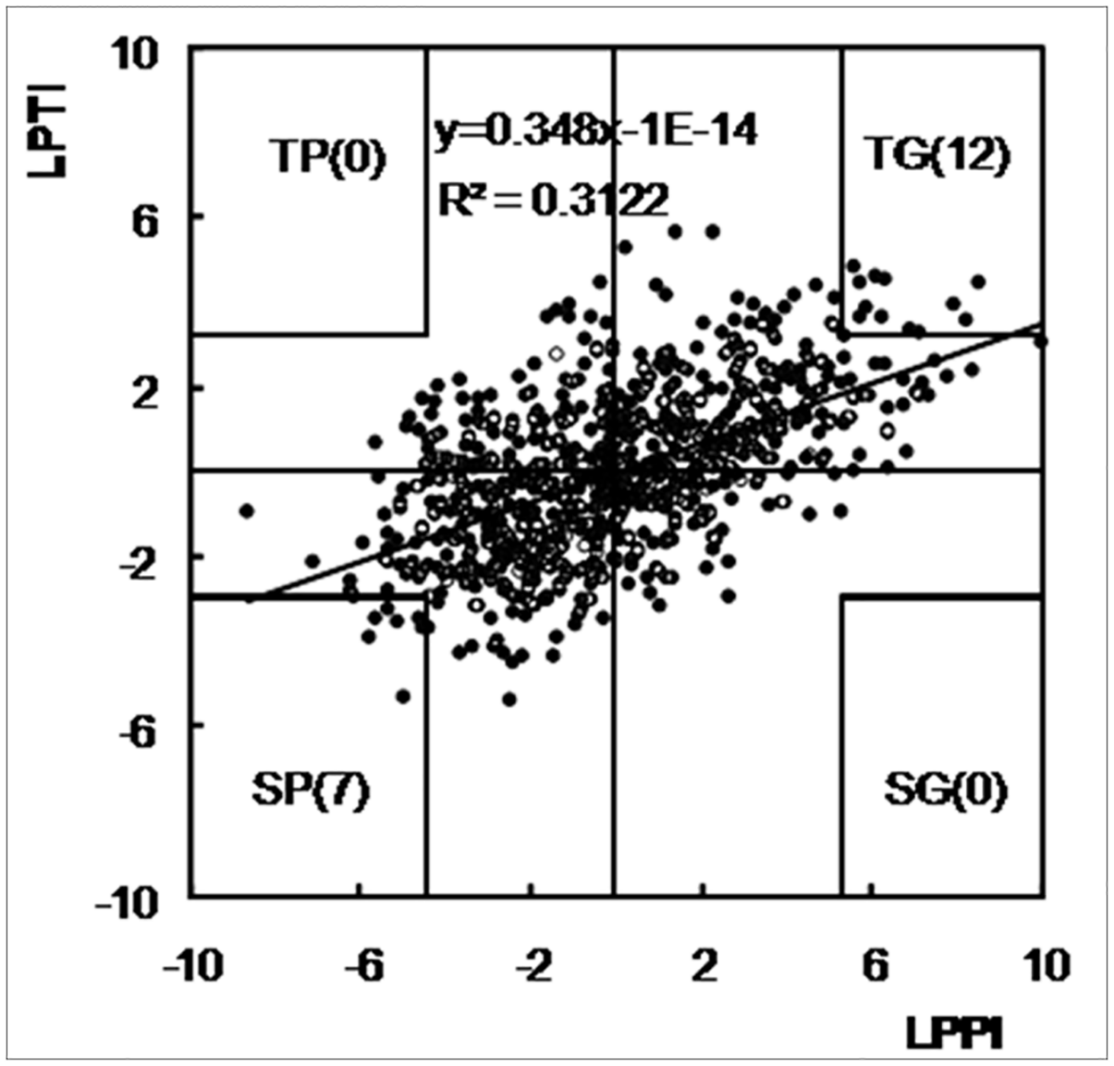
Classification of 826 maize accessions for their responses to LP stress based on LPTI and LPPI. LPPI: LP performance index; LPTI: LP tolerance index; TG: tolerant and good-performance group; SG: sensitive and good-performance group; TP: tolerant and poor-performance group; SP: sensitive and poor-performance group. The number in the bracketsis the number of maize accessions in each group.

The significant correlation between LPTI and LPPI (r = 0.56, P < 0.001) suggests that better trait performance under LP conditions would result into greater LP tolerance. The 826 accessions were further classified into four groups based on LPTI and LPPI. The midpoints between the value of 41^st^ and 42^nd^ accessions were taken as the dividing points for selecting the top and bottom ~5% of the four groups. Thus, the corresponding dividing points for selecting the top and bottom ~5% accessions were 5.32 and -4.44 for LPPI and 3.25 and -2.92 for LPT1. As shown in Fig 3, the coordinates of the dividing points in the first, second, third, and fourth quadrants were 5.32 and 3.25, -4.44 and 3.25, -4.44 and -2.92, and 5.32 and -2.92, respectively. Two lines that were horizontal to each of the two axes passed through each dividing point, producing four corners in the four quadrants. The extreme accessions in the TG, TP, SG, and SP groups corresponded to the points in the four corners (Fig 3).

Based on the procedure described above, the groups TG, TP, SG, and SP contained 12, 0, 7, and 0 accessions, respectively (Fig 3). The TG group not only showed tolerance to LP stress, but also performed better under LP conditions. The 12 accessions in the TG group included 11 and 1 lines from C6 and Trop populations, respectively (Fig 3; S2 Fig). The SP group was sensitive to LP stress and performed poorly under LP conditions (S2 Fig), the seven accessions in this group included 1, 5 and 1 accessions from C5, IL and Trop populations, respectively (S1 Table). TP group should have similar LP tolerance with TG, and poor performance under LP conditions, but no accession were identified in this group. Meanwhile, no accessions were identified in the SG group, which was supposed to be sensitive to LP stress and grow better under LP conditions.

## Discussion

It is imperative to select low-P tolerant maize germplasm resources to combat the threat of suboptimal soil P availability to world maize production [3]. However, only several maize germplsam evaluation experiments have been reported. Using 242 maize accessions, mostly landraces and synthetic hybrids, 41 and 98 accessions were classified as P-efficient and-inefficient based on the relative trait values of multiple field traits [26]. In the experiment conducted in Southeast China, 5 and 36 inbreds were identified as LP-tolerant and-sensitive ones among 76 inbreds using seedling traits collected in the field [20]. In another report, 23 and 109 LP-tolerant and-sensitive inbreds were identified out of 456 diverse inbreds based on seedling traits [20]. A total of 112 of the 456 inbreds were also used in this study, 31 and 12 accessions were classified as extreme accessions in the previous and present studies, respectively [20], and some extreme accessions were not shared between the two studies, which might be caused by the following reasons: (1) the extreme accessions identified in the previous study might not be classified to be extremely tolerant or sensitive owing to the larger population size used in this study; (2) the growth conditions between the present experiment (field) and previous report (hydroponic) are totally different; (3) the traits evaluated in this study were more comprehensive and more representative in terms of growth stages and trait groups [20]. Conclusively, the extreme accessions selected in this study should be more reliable and could be used to improve LP tolerance of maize.

### Comparison of selections with different LPTIs

It has been well documented that LP stress not only reduces leaf development but also retards growth in terms of biomass and PH [27, 28]. However, it has been seldom considered whether selection of LP tolerance is affected by different traits. In order to compare the selection results using biomass traits and leaf traits, the two trait groups were used to calculate LPTI_bm and LPTI_lf. Correlation analysis showed that LPTI_bm and LPTI_lf had strong correlation with the relative trait values that were used to calculate them (S2 Table), supporting that LPTI_bm and LPTI_lf could be used to represent biomass and leaf traits. Similarly, it is also important to compare the selection results obtained at different stages using LPTI_el and LPTI_lt, which showed strong correlation with the relative trait values calculated using early-stage and late-stage traits, respectively.

Our previous reports identified four consensus QTL controlling biomass and leaf traits under LP conditions on chromosomes 2, 3, 4 and 5 [29], indicating that the responses of the two types of traits to LP conditions might be genetically related. Consistently, the strong correlations between biomass traits and leaf traits (Table 3) and between LPTI_bm and LPTI_lf (r = 0.70, P < 0.001) also indicate that selection results for LP tolerance might be similar regardless of which kinds of traits were used. In this study, we compared the LP-tolerant and-sensitive maize accessions selected based on biomass traits and leaf traits, respectively. We noticed that, among the 41 sensitive and tolerant accessions, 13 sensitive accessions and 12 tolerant accessions were selected by both biomass and leaf traits (S3 Table). These results suggest that selection based on either of the two trait groups was generally reliable and that some common genetic factors were shared between LP tolerance indices measured by the two trait groups.

Previous reports suggested that some common chromosome regions controlled LP tolerance at different stages. For example, a QTL on chromosome 12 had a consistent effect on shoot P concentration from 40 to 150 days after sowing [30]. Nine consensus QTL controlling traits measured at different stages under LP conditions were also identified by QTL meta-analysis [29]. It follows that some common genetic factors might control LP tolerance at both early and late plant stages and that accessions with extreme phenotypes selected at an early stage might also have extreme phenotypes at a late stage. In this study, the correlation between LPTI_el and LPTI_lt was as high as 0.69, with 12 sensitive and 13 tolerant accessions (out of 41) shared between the two selections (S3 Table). Given that some tolerant and sensitive accessions showed consistent tolerance or sensitivity across different stages, breeders are advised to select at an early stage which would reduce the cost of selection by shortening the screening time and reducing the screening space.

### Implications of LPTI and LPPI

Understanding the mechanism for LP tolerance (LPTI) is an important goal of basic plant biology [3], and evaluating plant performance under LP conditions (LPPI) has practical importance for cultivar development [27]. Simultaneous selection for both LPPI and LPTI would identify four extreme groups (Fig 3). TG group showed tolerance and had better trait performance under LP conditions, whereas SP group was sensitive and showed poor performance under LP stress. TG and SP groups consisted of 12 and 7 accessions, respectively, suggesting that LP-tolerant/-sensitive germplasm could be easily found from accessions that had good/poor performance under LP conditions. The general trend that LPTI increased with the increase of LPPI also indicates that most LP-tolerant and-sensitive accessions could be identified from accessions that had good and poor performance under LP conditions, respectively (Fig 3), implying that trait performance under LP conditions was indicative of tolerance to LP stress. On the other hand, no accessions were found in the TP and SG groups, suggesting that LP-tolerant accessions could hardly be found from accessions that had poor performance under LP conditions, and *vice versa*. The accessions selected from TG and SP groups should be considered as the best parental lines for developing bi-parental populations for genetic mapping of LP tolerance, because the two groups represent not only the extreme phenotypes for LP tolerance but also the extreme phenotypes for good performance under LP conditions.

### Comparison of LP tolerance among different population types

Artificial selection during breeding has narrowed the genetic basis of crops including maize [31]. Identification of LP-tolerant germplasm from tropical/subtropical maize would be useful for enlarging the genetic variation of Chinese temperate maize. When comparing the extreme accessions contributed by five population types to, the tropical C5 RIL population was found to show slight bias toward LP sensitivity (S3 Table), which was consistent with the LP tolerance rankings of Ac7643 and Ac7729/TZSRW (S1 Table). Ac7643, which was characterized with developed root system, was more tolerant to both drought and LP stresses than Ac7729/TZSRW (S1 Table) [32]. This result indicates that root traits could be used as criteria for evaluation of maize germplasm for both LP and drought tolerance. Meanwhile, the reason why C5 RIL contributed more LP-sensitive accessions but C6 RIL contributed more LP-tolerant accessions needs to be further investigated with the availability of genotype data in the future.

Determining whether the tropical/subtropical or temperate ecotype is more tolerant to LP stress would be useful. Because favorable/detrimental genes controlling LP tolerance are shared among some lines derived from biparental populations, it is reasonable to exclude these lines and use only inbred lines to compare tropical/subtropical vs. temperate germplasm for LP tolerance. Therefore, this study used 304 inbred lines (217 tropical/subtropical and 87 temperate inbreds in comparison. The mean LPTI value was found to be smaller for the tropical/subtropical inbreds (-0.37) than that for the temperate (0.46). Meanwhile, the relative trait values of temperate inbreds were larger than those of the tropical/subtropical inbreds for 13 of the 14 traits (S3 Fig), which may indicate that the temperate inbreds showed slightly better tolerance to LP stress. Similar conclusions could also be found from a previous low-P tolerance evaluation [20]. However, for 15 inbreds selected as top 5% of the best LP tolerant inbreds from the 304 tested inbreds, 11 of them are tropical/subtropical while only four are temperate, which might be due to the fact that we tested more tropical/subtropical inbreds. We therefore suggest that accessions from both tropical/subtropical and temperate regions should contain LP-tolerant germplasm that are useful for improvement of LP tolerance in maize.

## Supporting Information

S1 Fig Confirmation of the selection results based on LPTI. Reduction rates of PH and LN were used to confirm the differences in LP tolerance among three groups (P-sensitive, P-tolerant, and others); Reduction rate = (trait value under NP condition—trait value under LP condition)×100/ trait value under NP condition. PH: plant height; LN: leaf number; NDVI: normalized difference vegetation index; FEW: fresh ear weight; DAP: days after planting; S-group included the 41 sensitive accessions; T-group included the 41 tolerant accessions; others included the remaining accessions.
(TIF)

S2 Fig Confirmation of the selection results based on LPPI and LPTI. All the 14 traits tested were used to demonstrate the differences among two groups (TG and SP); NDVI: normalized difference vegetation index; FEW: fresh ear weight; DAP: days after planting; TG: tolerant and good-performance group; SP: sensitive and poor-performance group.
(TIF)

S3 Fig Comparison of relative trait values for 14 traits between tropical/subtropical and temperate inbreds. PH: plant height; LN: leaf number; NDVI: normalized difference vegetation index; FEW: fresh ear weight; relative trait value for each trait was calculated following: trait measured under LP/trait measured under NP.
(TIF)

S1 Table The accessions used for LP tolerance screening and classification of their LP tolerance. Notes: a, Population type: the accessions could be classified into five population types, including C5 RIL, C6 RIL, IL, Trop and Temp; b, There are the two different ecotypes: Tropical/subtropical and temperate; c, Selection-1: selection based on LPTI, S—sensitive to LP stress, T—tolerant to LP stress; d, Selection-2: selection based on LPTI and LPPI, TG: tolerant and good-performance group; SP: sensitive and poor-performance group; e, the ranking of LP tolerance was based on LPTI value, and larger value means greater tolerance to LP stress.
(XLSX)

S2 Table Correlation between LPTIs and the relative trait values that were used to calculate the corresponding LPTIs. Notes: LPTIs refer to LPTI, LPTI_bm, LPTI_lf, LPTI_el, and LPTI_lt; Correlation coefficients > 0.07, 0.09 and 0.11 were significant at 0.05, 0.01 and 0.001 level, respectively; a. Correlation coefficients between LPTI and the relative trait value for each trait; b. Correlation coefficients between LPTI_bm and the relative trait value for biomass traits (shaded), and between LPTI_lf and the relative trait value for leaf number traits (underlined); c. Correlation coefficients between LPTI_el and the relative trait value for early-stage traits (in bold), and between LPTI_lt and the relative trait value for later-stage traits (in italics).
(PDF)

S3 Table Selection of extreme lines based on multiple criteria. Notes: Selection based LPTI (a), LPTI_bm (b), LPTI_lf (c), LPTI_el (d) and LPTI_lt (e); common-1: number of the maize accessions shared by selections based on LPTI_bm and LPTI_lf; common-2: number of the maize accessions shared by selections based on LPTI_el and LPTI_lt.
(PDF)
