# Supplementary figures and images for: Large-Scale Evaluation of Maize Germplasm for Low-Phosphorus Tolerance
Source: PLoS One. 2015 May 4;10(5):e0124212. doi: 10.1371/journal.pone.0124212 (PMC4418814; doi:10.1371/journal.pone.0124212)

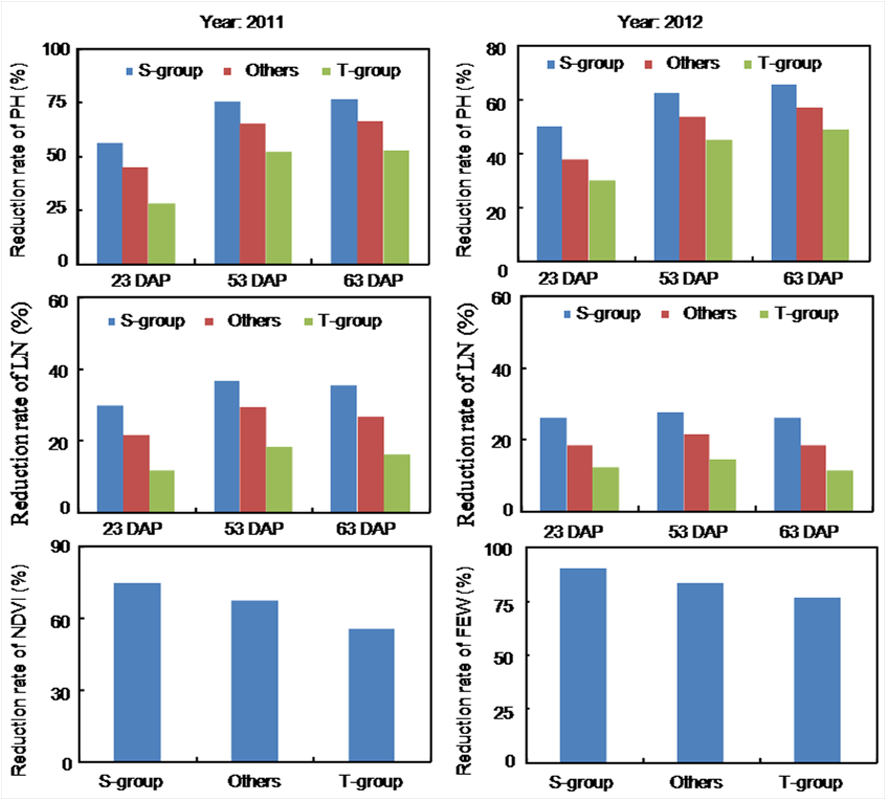

Supplement: S1 Fig — Reduction rates of PH and LN were used to confirm the differences in LP tolerance among three groups (P-sensitive, P-tolerant, and others); Reduction rate = (trait value under NP condition—trait value under LP condition)×100/ trait value under NP condition. PH: plant height; LN: leaf number; NDVI: normalized difference vegetation index; FEW: fresh ear weight; DAP: days after planting; S-group included the 41 sensitive accessions; T-group included the 41 tolerant accessions; others included the remaining accessions. (TIF) [file pone.0124212.s001.tif]

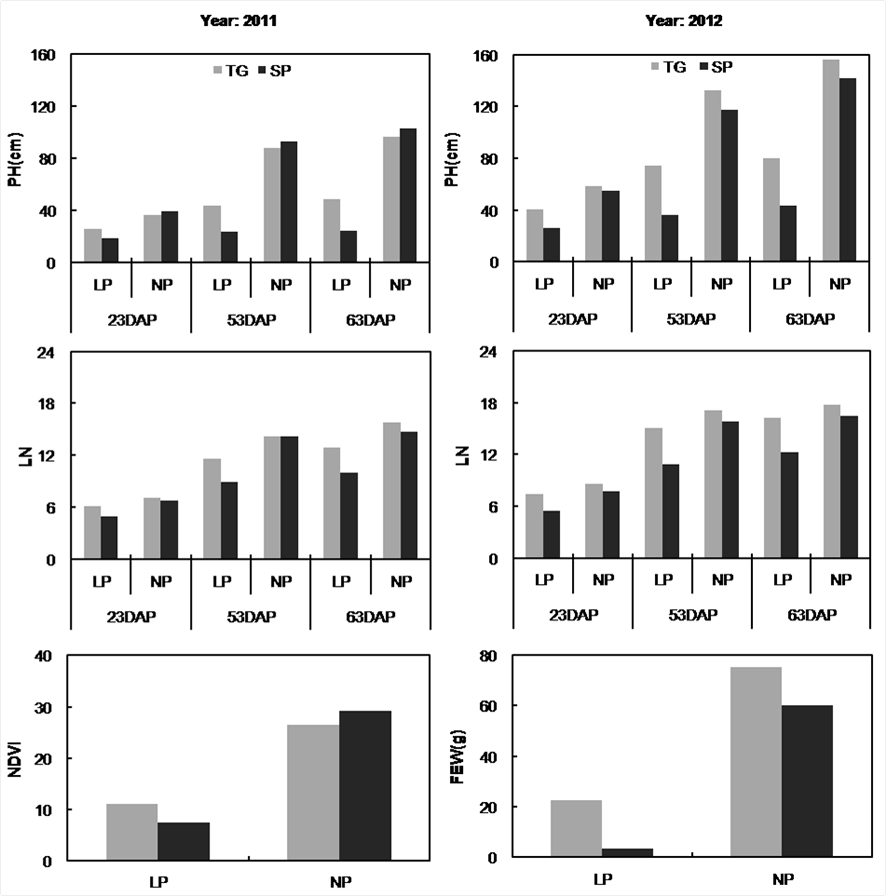

Supplement: S2 Fig — All the 14 traits tested were used to demonstrate the differences among two groups (TG and SP); NDVI: normalized difference vegetation index; FEW: fresh ear weight; DAP: days after planting; TG: tolerant and good-performance group; SP: sensitive and poor-performance group. (TIF) [file pone.0124212.s002.tif]

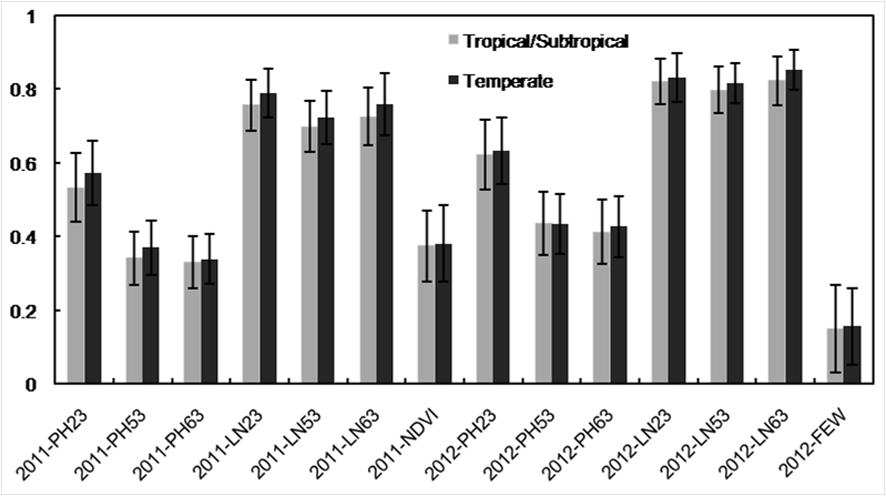

Supplement: S3 Fig — PH: plant height; LN: leaf number; NDVI: normalized difference vegetation index; FEW: fresh ear weight; relative trait value for each trait was calculated following: trait measured under LP/trait measured under NP. (TIF) [file pone.0124212.s003.tif]
