# Supplementary material for: Large-Scale Evaluation of Maize Germplasm for Low-Phosphorus Tolerance
Source: PLoS One. 2015 May 4;10(5):e0124212. doi: 10.1371/journal.pone.0124212 (PMC4418814; doi:10.1371/journal.pone.0124212)
Supplement: S2 Table — Notes: LPTIs refer to LPTI, LPTI_bm, LPTI_lf, LPTI_el, and LPTI_lt; Correlation coefficients > 0.07, 0.09 and 0.11 were significant at 0.05, 0.01 and 0.001 level, respectively; a. Correlation coefficients between LPTI and the relative trait value for each trait; b. Correlation coefficients between LPTI_bm and the relative trait value for biomass traits (shaded), and between LPTI_lf and the relative trait value for leaf number traits (underlined); c. Correlation coefficients between LPTI_el and the relative trait value for early-stage traits (in bold), and between LPTI_lt and the relative trait value for later-stage traits (in italics). (PDF) [file pone.0124212.s005.pdf]

S2 Table. Correlation between LPTIs and the relative trait values that were used to calculate the corresponding LPTIs

Traits	Year	Corr[a]	Corr[b]	Corr[c]
PH23	2011	0.63	0.65	**0.77**
	2012	0.55	0.52	**0.59**
PH53	2011	0.69	0.76	*0.71*
	2012	0.53	0.60	*0.57*
PH63	2011	0.68	0.77	*0.70*
	2012	0.55	0.62	*0.61*
VL23	2011	0.56	0.44	**0.74**
	2012	0.50	0.64	**0.61**
VL53	2011	0.62	0.58	*0.62*
	2012	0.57	0.78	*0.54*
VL63	2011	0.61	0.58	*0.60*
	2012	0.55	0.77	*0.53*
NDVI	2011	0.49	0.48	*0.51*
FEW	2012	0.31	0.40	*0.41*
