# Supplementary material for: Large-Scale Evaluation of Maize Germplasm for Low-Phosphorus Tolerance
Source: PLoS One. 2015 May 4;10(5):e0124212. doi: 10.1371/journal.pone.0124212 (PMC4418814; doi:10.1371/journal.pone.0124212)
Supplement: S3 Table — Notes: Selection based LPTI (a), LPTI_bm (b), LPTI_lf (c), LPTI_el (d) and LPTI_lt (e); common-1: number of the maize accessions shared by selections based on LPTI_bm and LPTI_lf; common-2: number of the maize accessions shared by selections based on LPTI_el and LPTI_lt. (PDF) [file pone.0124212.s006.pdf]

S3 Table. Selection of extreme lines based on multiple criteria

	Groups	C5	C6	IL	Trop	Temp	Total
Selection[a]	Sensitive	20	1	8	11	1	41
	Tolerant	2	24	4	8	3	41
Selection[b]	Sensitive	18	0	6	17	0	41
	Tolerant	3	28	4	4	2	41
Selection[c]	Sensitive	18	2	9	12	0	41
	Tolerant	4	15	2	13	7	41
Common-1	Sensitive	6	0	2	5	0	13
	Tolerant	1	7	1	2	1	12
Selection[d]	Sensitive	12	0	12	15	2	41
	Tolerant	2	28	2	5	4	41
Selection[e]	Sensitive	21	2	8	10	0	41
	Tolerant	3	21	5	9	3	41
Common-2	Sensitive	5	0	2	5	0	12
	Tolerant	0	9	2	1	1	13
